# German validation of Quality of Life after Brain Injury (QOLIBRI) assessment and associated factors

**DOI:** 10.1371/journal.pone.0176668

**Published:** 2017-05-24

**Authors:** Nicole von Steinbüchel, Ruben G. L. Real, Nadine Sasse, Lindsay Wilson, Christiane Otto, Ryan Mullins, Robert Behr, Wolfgang Deinsberger, Ramon Martinez-Olivera, Wolfgang Puschendorf, Werner Petereit, Veit Rohde, Holger Schmidt, Stephan Sehmisch, Klaus Michael Stürmer, Klaus von Wild, Henning Gibbons

**Affiliations:** 1 Institute of Medical Psychology and Medical Sociology, University Medical Center Goettingen, Goettingen, Germany; 2 Department of Psychology, University of Stirling, Stirling, United Kingdom; 3 Department of Child and Adolescent Psychiatry, Psychotherapy and Psychosomatics, University Medical Center Hamburg Eppendorf, Hamburg, Germany; 4 Department of Neurosurgery, Clinical Center Fulda, Fulda, Germany; 5 Department of Neurosurgery, Clinical Center Kassel, Kassel, Germany; 6 Department of Neurosurgery & Neurotraumatology at Bergmannsheil University Hospital Bochum, Bochum, Germany; 7 Westend Neurological Clinic, Bad Wildungen, Germany; 8 Department of Neurosurgery, Clinical Center Bernburg, Bernburg, Germany; 9 Department of Neurosurgery, University Medical Center Goettingen, Goettingen, Germany; 10 Department of Neurology, University Medical Center Goettingen, Goettingen, Germany; 11 Trauma surgery, plastic and reconstructive surgery, University Medical Center Goettingen, Goettingen, Germany; 12 KvW Neuroscience Consulting, Muenster, Germany; 13 Department of Psychology, University of Bonn, Bonn, Germany; Santa Lucia Fondation, ITALY

## Abstract

The consequences of traumatic brain injury (TBI) for health-related quality of life (HRQoL) are still poorly understood, and no TBI-specific instrument has hitherto been available. This paper describes in detail the psychometrics and validity of the German version of an internationally developed, self-rated HRQoL tool after TBI—the QOLIBRI (Quality of Life after Brain Injury). Factors associated with HRQoL, such as the impact of cognitive status and awareness, are specifically reported. One-hundred seventy-two participants after TBI were recruited from the records of acute clinics, most of whom having a Glasgow Coma Scale (GCS) 24-hour worst score and a Glasgow Outcome Scale (GOSE) score. Participants had severe (24%), moderate (11%) and mild (56%) injuries as assessed on the GCS, 3 months to 15 years post-injury. The QOLIBRI uses 37 items to measure “satisfaction” in the areas of “Cognition”, “Self”, “Daily Life and Autonomy”, and “Social Relationships”, and “feeling bothered” by “Emotions”and “Physical Problems”. The scales meet standard psychometric criteria (α = .84 to .96; intra-class correlation—ICC = .72 to .91). ICCs (0.68 to 0.90) and αs (.83 to .96) were also good in a subgroup of participants with lower cognitive performance. The six-subscale structure of the international sample was reproduced for the German version using confirmatory factor analyses and Rasch analysis. Scale validity was supported by systematic relationships observed between the QOLIBRI and the GOSE, Patient Competency Rating Scale for Neurorehabilitation (PCRS-NR), Hospital Anxiety and Depression Scale (HADS), Profile of Mood States (POMS), Short Form 36 (SF-36), and Satisfaction with Life Scale (SWLS). The German QOLIBRI contains novel information not provided by other currently available measures and has good psychometric criteria. It is potentially useful for clinicians and researchers, in post-acute and rehabilitation studies, on a group and individual level.

## Introduction

Health care and rehabilitation programmes following traumatic brain injury (TBI) aim to restore a person to everyday life with as high a quality of life (HRQoL) as possible. Accomplishing this poses remarkable challenges given that TBI can result in lifelong physical, cognitive, emotional, and behavioural impairments, as well as significant restrictions in social participation. Outcomes after TBI have traditionally been assessed using functional indicators, such as disability [1,2], health status [3–6], return to work or productivity [7–9], or psychosocial and social functioning [10–12]. While these outcomes are certainly linked to health-related quality of life (HRQoL) after TBI, they do not incorporate the specific subjective perspective of wellbeing and functioning, which are important aspects of HRQoL [13,14]. However, lately wellbeing and HRQoL have become important outcome variables after TBI: even patients after mild TBI (mTBI) show significantly reduced general wellbeing in a prospective study with a longitudinal design [15]. Very little literature exists on the assessment of wellbeing or HRQoL in German TBI samples. German studies have used the Aachen Life Quality Inventory [16] or the SF-36 [5,17], both of which are instruments for assessing generic HRQoL. However, when so assessed even individuals after mTBI display significantly reduced general wellbeing [18].

QoL is defined by the World Health Organization QOL Group [19] (p. 153) as”… an individual’s perception of their position in life in the context of the culture and value systems in which they live and in relation to their goals, expectations, standards, and concerns…”. HRQoL refers to the specific effects of health conditions on a person’s wellbeing and functioning. HRQoL measures are increasingly regarded as essential to elicit a more complete assessment of treatment effects and providing information concerning the patient’s subjective experience via self-reporting. Such patient-reported outcomes (PRO) therefore provide a measure of the subjective outcome and, furthermore, attempt to avoid significant clinician bias in outcome studies. Generic HRQoL instruments and measures of subjective health, such as the SF-36 [20] or the Sickness Impact Profile (SIP, [21]), do not cover specific areas that are relevant to TBI, for example, cognition or self-esteem, as well as future prospects. A disease-specific HRQoL instrument may be more sensitive to the particular consequences of TBI and more relevant to the patient. In response to the need for a disease-specific measure of HRQoL after TBI, an international multidisciplinary group was formed to develop the QOLIBRI descriptive system [14,22,23]. The instrument was constructed in a consensual fashion, in several languages simultaneously, and validated in two cross-sectional studies. The second international study included six language versions of the QOLIBRI (Dutch, English, Finnish, French, German, and Italian) and a total of 921 persons with TBI [22,23].

The purpose of this study is to examine the psychometric properties of the German language version of the QOLIBRI. Some information concerning the reliability and validity of different language versions has already been reported in the context of the international study [22,23], but there has been a lack of a detailed examination of individual language versions to show that their psychometric properties are appropriate for use.

As well as studying reliability and aspects of construct validity, we were particularly interested in whether confirmatory factor analysis in a single-language version, in this case German, would support the QOLIBRI’s descriptive system. The second aim of this study is to extend the validation of the QOLIBRI by examining relationships with measures that were not part of the international studies, namely measures of disability, mood states, and general wellbeing and satisfaction. Additionally, the generally little investigated issue of the influence of impaired cognition on self-reported HRQoL after brain injury is examined here in more detail.

## Materials and methods

### Participants

A total of 172 individuals (118 male) after TBI participated in the study. Patients were recruited from hospitals in the German communities of Goettingen, Fulda and Kassel (n = 166). Additionally, six individuals from a rehabilitation centre took part. The *inclusion criteria* were: ICD-10 diagnosis of TBI, 3 months to 15 years post-injury, aged 15 years or more at time of injury, outpatient status, aged 17 to 68 years at time of interview, and able and willing to give written informed consent. *Exclusion criteria* included a Glasgow Outcome Score-Extended (GOSE; [24]) score less than 3 (vegetative state); spinal cord injury; significant current or pre-injury psychiatric history; ongoing severe addiction; inability to understand, cooperate and answer; and having a terminal illness. In contrast to the two international developmental and validation studies reported elsewhere [22,23], a Glasgow Coma Scale (GCS) score [25] was not required for inclusion in the present study, however a GCS score was obtained for 90% of participants. The study was approved by the local ethical review board of the University Medical Center Göttingen (No. 26308) and conformed to the Declaration of Helsinki [26].

### Measures

The QOLIBRI (Quality of Life after Brain Injury) questionnaire with its 37 items generates a profile of HRQoL in the six domains: “Cognition” (7 items), “Self” (7 items), “Daily Life and Autonomy” (7 items), “Social Relationships” (6 items), “Emotions” (5 items), and “Physical Problems” (5 items), as well as a total score. The first four scales contain items requiring “satisfaction” judgements, whereas the last two scales ask for judgements about how “bothered” the person feels. Patient responses are assessed via a five-point Likert scale from “not at all” to “very”.

To measure wellbeing in terms of generic HRQoL, the Satisfaction with Life Scale (SWLS; [27]) and the Short Form-36 (SF-36, [20]) were administered. In its usual form, the SWLS has seven-point Likert response scales. In the present study, five-point scales were used in order to simplify the scale for patients with cognitive deficits (“I disagree completely” to “I agree completely”). SF-36 Physical (PCS) and Mental (MCS) component summary scores were calculated using US normative data. The global functional status was assessed using the Glasgow Outcome Scale-Extended (GOSE). Interviewers were trained to administer the GOSE based on the manual [24]. Disabilities and competencies were investigated by means of the Patient Competency Rating Scale for Neurorehabilitation (PCRS-NR) [28], providing a total score, as well as three subscale scores (Emotional Functioning, Interpersonal Functioning, Cognitive Functioning).

The Hospital Anxiety and Depression Scale (HADS; [29]) served as a measure of emotional distress and symptoms of the participants. Depression and anxiety were categorised using conventional cut-offs (i.e. 0–7 represented the normal range, 8–10 mild, 11–14 moderate, and 15 or above severe depression or anxiety) [29]. Mood was screened in this study using a short version of the Profile of Mood States (POMS, [30]). This instrument has been validated in the German language and consists of 35 items on four subscales (Depression/Dejection, Vigour/Activity, Fatigue/Inertia, and Anger/Hostility).

The Telephone Interview for Cognitive Status (TICS; [31]) was administered to screen the cognitive status of the participant. A cut-off of 32/33 was used to define two groups, having low cognitive performance and normal performance.

Socio-demographic data, including age, gender, education, employment, profession, living situation, autonomy, leisure interests and activities, aspects of social life, alcohol and nicotine consumption, as well as participation in rehabilitation, were assessed using a questionnaire that was completed by the participants [32].

Participants’ current health conditions were self-reported on a health symptoms list of 28 health conditions (adapted from the WHO-QoL project; [32]). For severity of injury, the 24-hour worst Glasgow Coma Scale (GCS; [25]) score was used, a standard index for severity classification, whereby scores of 3 to 8 indicate severe injury, 9 to 12 moderate injury, and 13 to 15 indicate mild injury. Comorbid health problems, such as epilepsy, hemiparesis, vision and hearing problems, extra-cerebral injuries, communication, attention and memory dysfunction, executive function, and affective and behavioural disorders, as well as participation in rehabilitation and medications, were also noted.

### Data collection

The GOSE and TICS were administered as a telephone interview. During this interview, a clinical checklist was also completed that covered the inclusion and exclusion criteria for the study as well as comorbid health conditions. The 24-hour worst GCS score was obtained from the patients’ medical records. These records also provided information concerning major brain lesions and the date of injury. HRQoL was assessed via self-rated questionnaires.

Two methods were used for data collection: a) patients received and returned the questionnaire by post and were interviewed by telephone (N = 168) or b) patients visited the clinic to complete the questionnaire and were interviewed in a face-to-face meeting (N = 4). The QOLIBRI was assessed at two points in time, i.e. test and retest, with an average interval of two weeks.

### Statistical analyses

We followed the approach used in the international sample, as described in detail elsewhere [22,23]. Data were analysed using PASW 18 software. Item scores on the “bothered” QOLIBRI scales “Emotions” and “Physical Problems” were recoded to match scores on the “satisfaction” scales. Missing values were prorated if less than 33% of the answers per scale were missing, and mean percentage scores were calculated for the six QOLIBRI subscales and the QOLIBRI total.

The analyses included descriptive and confirmatory analyses. The psychometric analyses focused on I) factorial validity of the QOLIBRI in the German sample, II) reliability and III) construct validity, and IV) partial correlations.

1. Classical as well as modern test-theoretical approaches were applied. Rasch analysis, Principal Components Analysis (PCA), and Confirmatory Factor Analysis (CFA) using methods based on Structural Equation Modelling (SEM) served to investigate the factorial validity of the QOLIBRI. Rasch analyses were conducted using the Winsteps 3.66 implementation of a partial credit model [33]. Item fit to a Rasch model was examined using ‘infit’ and ‘outfit’ statistics: values between 0.70 and 1.30 are regarded as acceptable for rating scales. CFA was carried out using Amos 18. Likewise, Promax rotation in PCA was used, and a second-order CFA model was specified, including a higher order factor besides the six latent variables representing the six QOLIBRI scales. To describe how well our model fits the data, recommended fit indices were chosen which are relatively independent of sample size [34]. The Root Mean Square Error of Approximation (RMSEA; [35]) served to describe overall fit, while the incremental Comparative Fit Index (CFI; [36]) compared our model to a baseline model. In addition, the Standardized Root Mean Square Residual (SRMR; [37]) focuses on residuals unexplained by the specified model. The following “rules of thumb” were used for the fit indices: an RMSEA ≤ 0.05 indicates a close fit, while a value ≤ 0.08 represents an acceptable fit [38]. For the CFI, a value ≥ 0.97 stands for a close fit, but a CFI ≥ 0.95 represents an acceptable fit [34]. Finally, an SRMR ≤ 0.05 indicates a close fit and values ≤ 0.1 represent acceptable one [39].
2. The internal consistency of the QOLIBRI was analysed by calculating Cronbach’s α for each scale using data from the first and second time points. Cronbach’s α ≥ 0.70 indicates acceptable reliability for measures used in group comparisons [40], while measures applied to individuals in clinical settings should reach values ≥ 0.90 [41]. To investigate the test-retest reliability, we calculated the intra-class correlation coefficient (ICC). ICCs between 0.40 and 0.75 are usually interpreted as fair to good, while ICCs > 0.75 indicate excellent test-retest reliability [42]. To analyse the reliability of the QOLIBRI in relation to the cognitive status of participants, Cronbach’s α and ICC were calculated for subgroups of patients defined by the TICS using a recommended cut-off (i.e. a sum score < 33 indicates a low cognitive status, while a score > 32 represents a normal cognitive status; [43].
3. The analysis involved a mixture of continuous and ordinal measures, and some skewness was present in the majority of variables. Unless otherwise noted, analyses were carried out on ranked variables [44], and Spearman correlations were used to examine relationships between HRQoL and other variables.
4. Partial correlations were used to assess whether the QOLIBRI total score contained information not included in other measures.

## Results

### Sample characteristics

The sample consisted of 172 patients with TBI who met the inclusion criteria and gave their informed consent to participate in this study; they completed the QOLIBRI questionnaire and were interviewed by telephone for the GOSE and TICS. In most cases, a telephone interview was conducted, followed by the collection of self-reported data by post (98%). The response rate for the QOLIBRI retest was 76% (i.e. all study participants were asked to take part in the retest, and 130 individuals responded). Table 1 provides a description of the sample.

**Table 1:** Characteristics of the sample (N = 172).

	Group	Frequency (rate)
**Sex**	Male	118 (69%)
	Female	54 (31%)
**Age**	17 to 30 years	34 (20%)
	31 to 44 years	35 (20%)
	45 to 68 years	103 (60%)
**Employment status**	Full-time	67 (39%)
**Relationship status**	Single	34 (20%)
	Partnered	120 (70%)
	Past partnered	17 (10%)
**Living arrangements**	Independent	123 (72%)
	Supported	48 (28%)
**Years since injury**	<1 year	20 (12%)
	1 to <2 years	24 (14%)
	2 to < 4 years	53 (31%)
	4 to 15 years	75 (44%)
**Major lesion**	None	37 (22%)
	Focal	113 (66%)
	Diffuse	6 (4%)
**Glasgow Coma Scale (worst score, first 24 hrs.)**	Severe (3–8)	40 (24%)
	Moderate (9–12)	18 (11%)
	Mild (13–15)	97 (56%)
**Glasgow Outcome Scale—Extended score**	Severe disability	20 (12%)
	Moderate disability	60 (35%)
	Good recovery	92 (54%)
**Number of comorbid health conditions**	0 to 3	71 (41%)
	4 to 6	40 (23%)
	7 or more	61 (36%)
**HADS**^1^ **- anxiety**	Normal (0–7)	125 (73%)
	Mild (8–10)	19 (11%)
	Moderate (11–14)	19 (11%)
	Severe (15–21)	9 (5%)
**HADS—depression**	Normal (0–7)	123 (72%)
	Mild (8–10)	19 (11%)
	Moderate (11–14)	25 (15%)
	Severe (15–21)	5 (3%)
**TICS**^2^ **- cognitive status**	Low	77 (45%)
	Normal	95 (55%)

^1^ Hospital Anxiety and Depression Scale;

^2^ Telephone Interview for Cognitive Status.

More than half of the participants were in the mild range on the GCS and more than half of the sample achieved a good recovery on the GOSE. In addition, more than 70% of the patients were living independently; while on the HADS 73% were classified as below the threshold for anxiety and 72% below the threshold for depression. Moreover, 45% of the sample had a low cognitive status on the TICS.

### Factorial validity

Less than 5% of the responses on QOLIBRI items were missing at initial testing (e.g. satisfaction with sexual life; 4.1%). However, 10% of the patients did not answer the QOLIBRI item concerning “participation in work or education”. This can be explained by the fact that in general this question does not apply to persons who have retired (12 out of 17 of these did not respond). After prorating, the QOLIBRI data collected at the first time point and at the retest were complete for each patient. However, we detected a significant deviation from a normal distribution for the QOLIBRI items (i.e. many items were skewed). Therefore, the ranked items were used in the analysis.

A Rasch analysis of each QOLIBRI subscale in turn indicated an acceptable fit for the majority of items. Minor deviations of infit were observed for two items: “Way you look” (1.34) and “See/hear” (1.36). Deviations of outfit were also found for two items: “Decisions” (1.31) and “See/hear” (1.35). Scale person reliabilities ranged from .81 to .90 for the first four scales, and were lower for the last two scales, “Emotions “(.63) and “Physical Problems” (.69). The interpretation of these reliability statistics is similar to Cronbach’s alpha, and indicates that the last two scales are somewhat weaker than the first four and show poorer discrimination between persons.

Two PCAs were conducted, forcing either one or six components (see Table 2).

**Table 2:** Principal components analyses of QOLIBRI items. Loadings < 0.25 are suppressed.

QOLIBRI scale	Item	1^st^ component	h^2^	Factor 1	Factor 2	Factor 3	Factor 4	Factor 5	Factor 6
**Cognition**	Concentrate	0.719	0.693		**0.685**			0.253	
	Express yourself	0.574	0.601		**0.791**				
	Remember	0.565	0.662		**0.945**				
	Plan and problem solve	0.667	0.684		**0.801**				
	Decisions	0.723	0.630	0.400	**0.451**				
	Find way	0.712	0.635		**0.614**				
	Speed of thinking	0.730	0.691		**0.723**				
**Self**	Energy	0.753	0.624						**0.438**
	Motivation	0.736	0.589	**0.299**					0.256
	Self-esteem	0.748	0.718						**0.656**
	Way you look	0.582	0.547						**0.683**
	Achievements	0.702	0.639						**0.621**
	Self-perception	0.755	0.707						**0.640**
	Own future	0.715	0.564						**0.374**
**Daily life & autonomy**	Independence	0.721	0.723	**0.809**					
	Get out & about	0.705	0.707	**0.863**					
	Domestic activities	0.619	0.582	**0.834**					
	Run personal finances	0.621	0.540	**0.688**					
	Participation work	0.744	0.617	**0.546**					
	Social&leisure activities	0.737	0.661	**0.601**					
	In charge of life	0.758	0.703	**0.762**					
**Social relation-ships**	Affection towards others	0.607	0.700			**0.780**			
	Family	0.615	0.647			**0.667**			
	Friends	0.645	0.625		**0.369**	**0.367**	0.333		
	Partner	0.431	0.738			**0.901**			
	Sex life	0.566	0.571			**0.695**			
	Attitudes of others	0.659	0.581	0.257		**0.530**			
**Emotions**	Loneliness	0.592	0.715				**0.803**		
	Boredom	0.410	0.604				**0.858**		-0.289
	Anxiety	0.584	0.635				**0.743**		
	Depression	0.670	0.732				**0.690**		0.327
	Anger/aggression	0.527	0.501				**0.624**		
**Physical problems**	Slow/clumsiness	0.666	0.736					**0.709**	
	Other injuries	0.405	0.607					**0.776**	
	Pain	0.496	0.552				0.320	**0.624**	
	See/hear	0.351	0.579					**0.708**	-0.396
	TBI effects	0.635	0.735					**0.651**	

Table 2 shows a one-factor solution and a six-factor solution. In the one-factor solution (*R*^*2*^ = .41) all QOLIBRI items had a satisfactory loading (>0.40), except for item “problems in seeing and/or hearing”. In the six-factor solution (*R*^*2*^ = .64), all but two QOLIBRI items had their highest loading on the appropriate scale, the number of cross-loadings (>.25) was low, and all factors had an *eigenvalue* > 1.0. The “Motivation” item had its highest loading on the factor representing “Daily Life and Autonomy” scale, while the loadings for the item “Friends” were nearly identical on the factors corresponding to the “Social Relationships” and “Cognition” scales.

**Fig 1:**
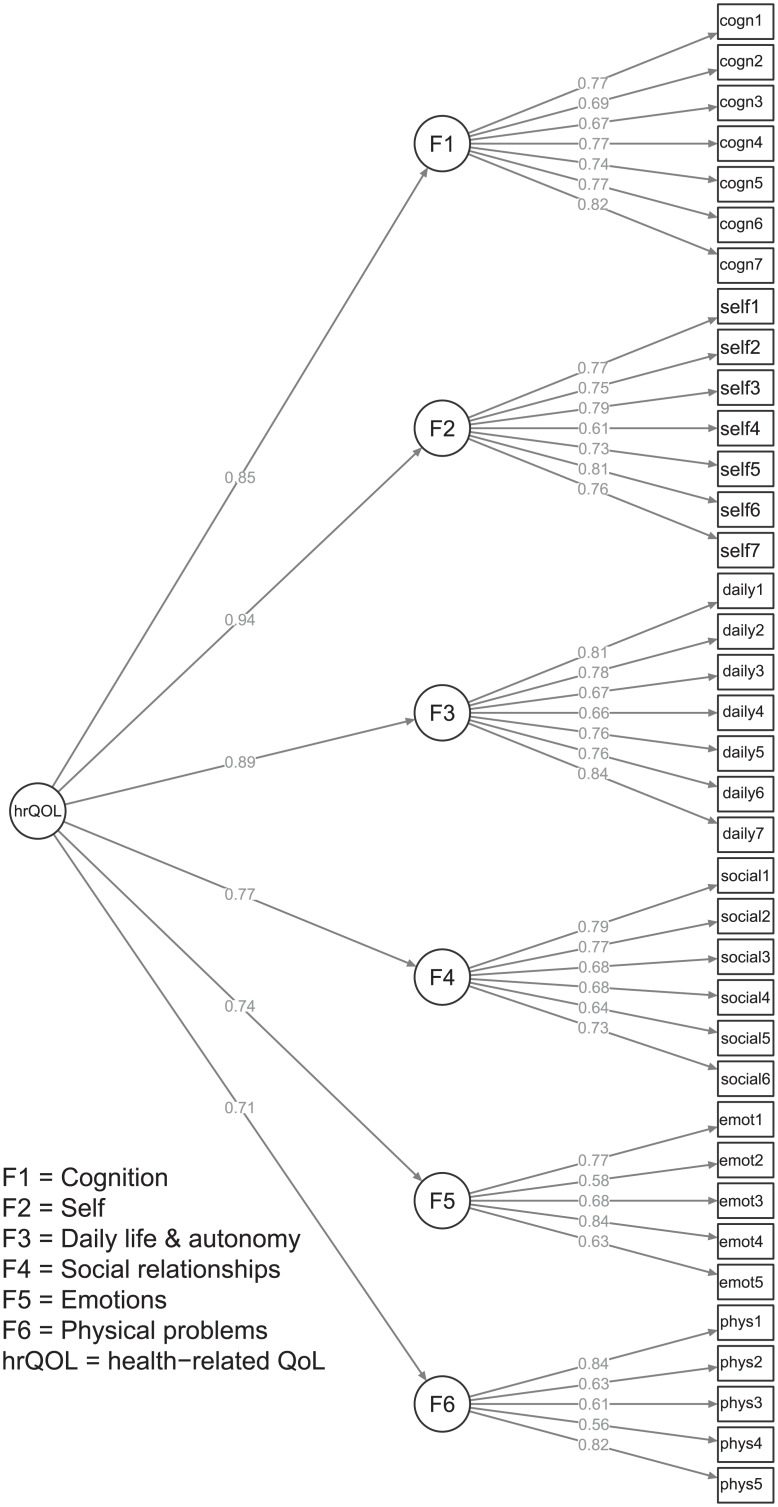
Path diagram of the second-order model of the QOLIBRI.

Moderate to high intercorrelations of the six QOLIBRI scales (Table 3) suggest a second-order CFA model, which is in line with the concept of an overall dimension of QoL representing subjective wellbeing. The second-order CFA model was specified (see Fig 1) and tested (Chi^2^ = 1,007, df = 623, RMSEA = 0.060, SRMR = 0.070 and CFI = 0.897). Our model showed an acceptable overall fit on RMSEA, but the CFI for our model indicated lack of fit. On the other hand, the SRMR showed an acceptable fit of the model with respect to residuals. The loadings of the first-order factors on the second-order factor were high, ranging from .71 to .91. As is common in SEM-based second-order CFA models, measurement errors for each indicator/item were included in the model (i.e. e1 to e37) and a residual term was estimated for each first-order factor (i.e. d1 to d6).

**Table 3:** Inter-correlation of QOLIBRI scales.

QOLIBRI scales	Cognition	Self	Daily life & autonomy	Social relation-ships	Emotions
**Self**	0.72*				
**Daily life & autonomy**	0.67*	0.72*			
**Social relationships**	0.51*	0.69*	0.57*		
**Emotions**	0.49*	0.60*	0.54*	0.62*	
**Physical problems**	0.54*	0.55*	0.64*	0.36*	0.47*

Pearson correlations shown;

* p<0.001

### Reliability

Cronbach’s αs for the QOLIBRI scales are shown in Table 4.

**Table 4:** Internal consistencies at test and retest for QOLIBRI scales for all participants, and for groups with low or normal cognitive status.

	All				Low TICS^1^				High TICS			
	n	Cronbach’s α at test	n	Cronbach’s α at retest	n	Cronbach’s α at test	n	Cronbach’s α at retest	n	Cronbach’s α at test	n	Cronbach’s α at retest
**Cognition**	172	0.91	130	0.92	77	0.90	64	0.90	95	0.89	66	0.93
**Self**	172	0.90	130	0.91	77	0.90	64	0.89	95	0.89	66	0.91
**Daily life & autonomy**	172	0.90	130	0.92	77	0.89	64	0.91	95	0.89	66	0.91
**Social relationships**	172	0.85	130	0.85	77	0.85	64	0.81	95	0.86	66	0.88
**Emotions**	172	0.84	129	0.86	77	0.83	63	0.86	95	0.82	66	0.80
**Physical problems**	172	0.84	130	0.81	77	0.84	64	0.79	95	0.81	66	0.82
**QOLIBRI total**	172	0.96	130	0.97	77	0.96	64	0.96	95	0.96	66	0.97

^**1**^ Telephone Interview for Cognitive Status.

Similarly, the ICCs indicate a good to excellent test-retest reliability (Table 5). Analyses additionally revealed that the QOLIBRI is reliable for TBI patients with low as well as a normal cognitive status (see Tables 4 and 5; note that these analyses are based on unranked data).

**Table 5:** Test-retest intra-class correlations for all retested participants, and for groups with low or normal cognitive status.

	All				Low TICS^1^				High TICS			
	n	ICC^2^	Lower 95% CI^3^	Upper 95% CI	N	ICC	Lower 95% CI	Upper 95% CI	n	ICC	Lower 95% CI	Upper 95% CI
**Cognition**	130	0.85	0.80	0.89	64	0.84	0.75	0.90	66	0.83	0.74	0.89
**Self**	130	0.87	0.83	0.91	64	0.85	0.76	0.91	66	0.88	0.82	0.93
**Daily life & autonomy**	130	0.87	0.82	0.90	64	0.87	0.79	0.92	66	0.85	0.77	0.91
**Social relationships**	130	0.77	0.69	0.83	64	0.72	0.58	0.82	66	0.82	0.72	0.88
**Emotions**	129	0.72	0.63	0.80	63	0.68	0.52	0.79	66	0.72	0.58	0.82
**Physical problems**	130	0.91	0.88	0.94	64	0.90	0.84	0.94	66	0.92	0.87	0.95
**QOLIBRI total**	130	0.91	0.87	0.94	64	0.89	0.83	0.93	66	0.91	0.86	0.94

^**1**^ Telephone Interview for Cognitive Status;

^2^ intra-class correlation;

^3^ confidence interval.

### Construct validity

The only gender difference found was for the “Cognition” scale (p = 0.022), where the mean for women was slightly higher (mean = 88.18; SD = 43.84) than for men (mean = 85.73; SD = 52.33). For age, weak correlations were observed for the “Cognition” (r = -0.166; p = 0.030), “Daily Life and Autonomy” (r = -0.235; p = 0.002), “Physical Problems” (r = -0.235; p = 0.002), and the total QOLIBRI scales (r = -0.159; p = 0.037), indicating a decline in HRQoL with age. None of the other QOLIBRI scales had significant correlations with age.

Table 6 depicts relationships between the QOLIBRI scales, injury severity and outcome variables. The relationships shown in Table 6 were previously examined in the international study [22,23], and are included here for comparison with the larger group. HRQoL was not significantly correlated to severity of injury on the GCS, but significant positive associations were found with the GOSE for all QOLIBRI scales. HRQoL was related both to the amount of help needed in five activities of daily living and to the number of comorbid health conditions. TBI-specific HRQoL was significantly and negatively associated with HADS anxiety as well as HADS depression for each QOLIBRI scale.

**Table 6:** Relationships between QOLIBRI scales and GCS, GOSE, HADS, and SF-36.

	Cognition	Self	Daily life & autonomy	Social relationships	Emotions	Physical problems	QOLIBRI total
**GCS**	0.12	0.04	0.12	0.01	<-0.01	0.14	0.08
**GOSE**	0.45***	0.38***	0.52***	0.17*	0.26**	0.61***	0.50***
**Help needed**	-0.41***	-0.42***	-0.63***	-0.30***	-0.36***	-0.51***	-0.54***
**Comorbid health conditions**	-0.46***	-0.52***	-0.50***	-0.34***	-0.50***	-0.67***	-0.61***
**HADS anxiety**	-0.60***	-0.66***	-0.54***	-0.43***	-0.69***	-0.51***	-0.71***
**HADS depression**	-0.59***	-0.78***	-0.67***	-0.67***	-0.63***	-0.49***	-0.79***
**SF-36 PCS**	0.44***	0.42***	0.55***	0.17*	0.28***	0.74***	0.54***
**SF-36 MCS**	0.46***	0.64***	0.48***	0.65***	0.70***	0.37***	0.66***

* p<0.05,

** p<0.01,

*** p<0.001

GCS = Glasgow Coma Scale, GOSE = Glasgow Outcome Scale-Extended, HADS = Hospital Anxiety and Depression Scale PCS = Physical Component Summary; MCS = Mental Component Summary.

Positive correlations were found between SF-36 scales and each of the QOLIBRI scales (Table 6), indicating that a higher HRQoL is related to a better subjective health status in the Physical as well as Mental Component Scores. The SF-36 PCS was strongly correlated with the QOLIBRI “Physical Problems” scale, while the association with the “Social Relationships” scale was rather low. On the other hand, the SF-36 MCS was most closely associated with the QOLIBRI “Emotions” scale, while the relationship with “Physical Problems” was weaker.

**Fig 2:**
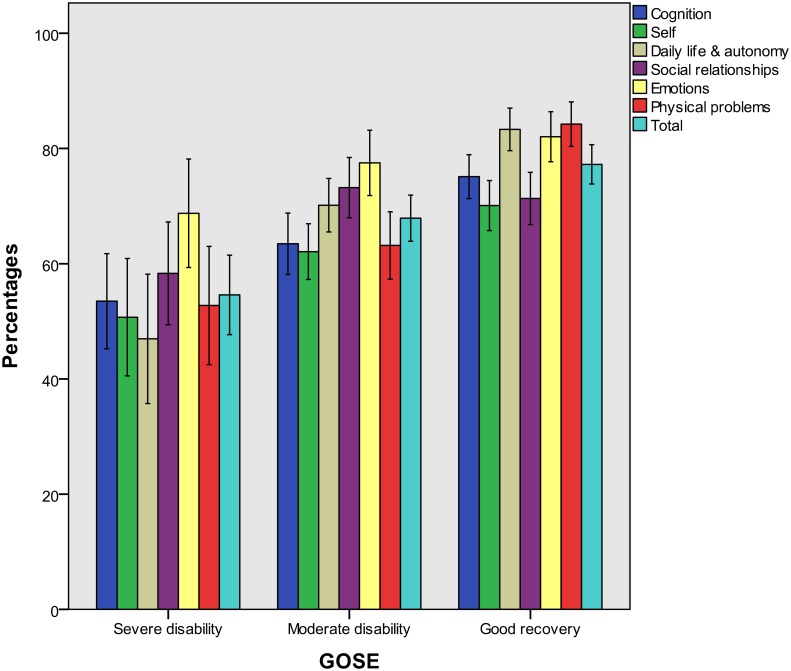
QOLIBRI scale percentage scores (means and 95% confidence intervals) for GOSE groups.

**Fig 3:**
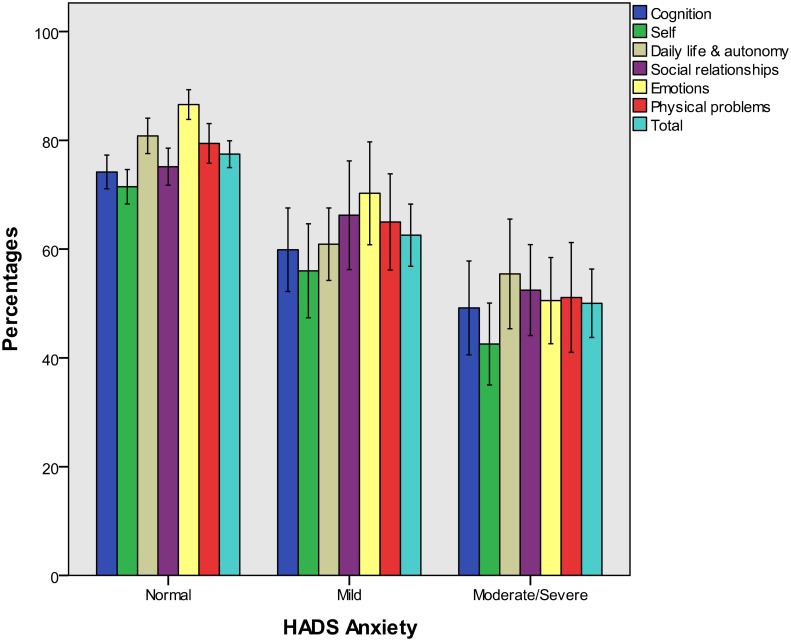
QOLIBRI scale percentage scores (means and 95% confidence intervals) for HADS anxiety groups.

**Fig 4:**
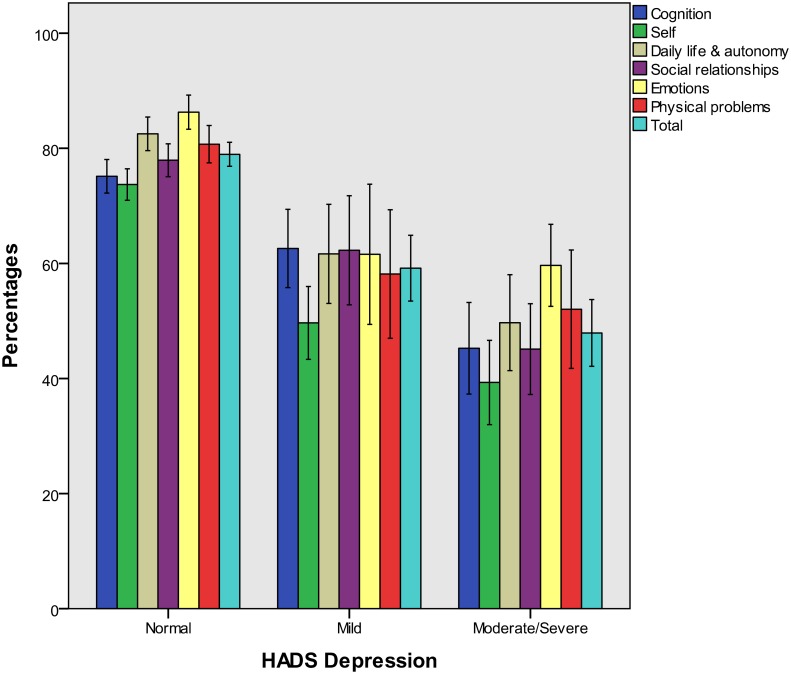
QOLIBRI scale percentage scores (means and 95% confidence intervals) for HADS depression groups.

Figs 2 to 4 present QOLIBRI percentage scores for groups of patients categorized by the GOSE and the HADS. In the present study, a small number of patients suffered from severe anxiety and an even smaller number from severe depression, as assessed on the HADS (i.e. < 10% in both cases). Therefore, these categories were collapsed for both variables, assigning moderate and severe cases to one group each for the anxiety and depression scales. Group-specific percentage scores on the QOLIBRI scales underline the validity of the QOLIBRI.

These results indicate that persons with a severe disability rate their HRQoL significantly worse on all dimensions in comparison to those with good recovery. Also, those with moderate disability experience a significantly worse HRQoL in all scales except for the ‘Social’ and ‘Emotions’ scales.

Table 7 shows the relationship between the QOLIBRI scales and measures that were included for the German sample and not described in studies already published. Cognition was assessed using both the TICS and clinician ratings. Significant correlations were found between the TICS and all QOLIBRI scales except the “Social Relationships” scale (see Table 7). The correlations with the TICS indicate that cognitive status is associated with HRQoL particularly on the “Cognition” and “Daily Life and Autonomy” scales. Clinician ratings of cognitive problems are also related to patient-reported HRQoL (Table 7). The ratings of attention and memory dysfunction generally appear to show the strongest relationships with QOLIBRI scales, particularly with the “Cognition” scale, the “Physical Problems” scale, and the QOLIBRI total.

**Table 7:** Relationships between QOLIBRI scales and measures of cognition, disability, emotional status, and wellbeing.

	Cognition	Self	Daily life & autonomy	Social relationships	Emotions	Physical problems	QOLIBRI total
**TICS**	0.38***	0.31***	0.43***	0.13	0.32***	0.33***	0.41***
**Clinician Ratings:**-							
Communication problems	0.36***	0.25***	0.27***	0.25***	0.16*	0.23**	0.33***
Attention dysfunction	0.46***	0.34***	0.29***	0.22**	0.30**	0.41***	0.42***
Memory dysfunction	0.38***	0.24**	0.31***	0.12	0.17*	0.39***	0.33***
Executive function disorders	0.28***	0.20**	0.23**	0.12	0.23**	0.22**	0.29***
Affective and behavioural Disorders	0.22**	0.20**	0.24**	0.16*	0.25**	0.32***	0.28***
**PCRS**:-							
Emotional functioning	0.55***	0.60***	0.50***	0.53***	0.64***	0.42***	0.66***
Interpersonal functioning	0.64***	.58***	.50***	.51***	.60***	.43***	.68***
Cognitive functioning	0.65***	0.46***	0.49***	0.30***	0.40***	0.37***	0.56***
PCRS total	0.71***	0.65***	0.58***	0.54***	0.67***	0.49***	0.75***
**POMS**:-							
Depression/ dejection	-0.54***	-0.72***	-0.59***	-0.58***	-0.68***	-0.38***	-0.72***
Vigour/ activity	0.54***	0.59***	0.52***	0.45***	0.49***	0.41***	0.62***
Fatigue/ inertia	-0.49***	-0.63***	-0.55***	-0.55***	-0.55***	-0.47***	-0.65***
Anger/ hostility	-0.36***	-0.50***	-0.36***	-0.46***	-0.52***	-0.33***	-0.50***
**SWLS**	0.52***	0.72***	0.66***	0.64***	0.57***	0.46***	0.74***

TICS = Telephone Interview for Cognitive Status; PCRS = Patient Competency Rating Scale; POMS = Profile of Mood States; SWLS = Satisfaction With Life Scale.

* p<0.05,

** p<0.01,

*** p<0.001

In this German sample, the relationship between self-rated competency/disability and HRQoL after TBI was investigated using the patient version of the PCRS-NR. Robust correlations are seen between the PCRS-NR and all QOLIBRI scales, indicating a strong link between HRQoL and self-rated competence in the three domains measured by this instrument (Table 7). Strong relationships are observed between “Emotional Functioning” and the “Emotions” scale, and between “Cognitive Functioning” and the “Cognition” scale. More generally, strong correlations (all r > = 0.49) are present between the PCRS-NR total and the individual QOLIBRI scales. The relationship between the PCRS-NR total score and the QOLIBRI total score is particularly strong (r = 0.75).

The POMS was included as an assessment of mood states, in addition to the HADS scales. As might be expected, there is a strong correlation between all POMS scales and the QOLIBRI. The strongest relationships between specific scales are between the POMS Depression/dejection scale and the QOLIBRI “Self”, “Emotions”, and total scales.

Correlations between the QOLIBRI scales and the SWLS sum score are shown in Table 7. Significant correlations for all QOLIBRI scales indicate that TBI-specific HRQoL and general wellbeing are closely related. The SWLS shows a particularly strong correlation with the QOLIBRI total score (r = 0.74), and the question arises whether there is any difference in the constructs that are being measured.

We studied the overlap between the QOLIBRI total score and the SWLS in more detail using partial correlations. The correlation between the GOSE total score and the QOLIBRI total scale, controlling for the SWLS, remained highly significant (r = 0.35; p<0.001), whereas the correlation between SWLS and GOSE was no longer significant after controlling for the QOLIBRI total score (r = 0.03; p = 0.695). These results indicate that the QOLIBRI provides information which would be lost if solely the SWLS was used. Differences between the SWLS and the QOLIBRI are also seen in the partial correlations using the HADS anxiety scale. The correlation between the HADS anxiety scale score and the QOLIBRI total scale, controlling for the SWLS, was highly significant (r = -0.56; p<0.001), but the correlation between the SWLS and the HADS anxiety scale was not significant when controlling for the QOLIBRI total score (r = 0.90; p = -0.010). However, for the HADS depression scale both partial correlations reached significance (r_SWLS to Depression controlling for QOLIBRI_ = -0.32; p<0.001; r_SWLS to Depression controlling for QOLIBRI_ = -0.55; p<0.001).

## Discussion

Brain injuries are not only associated with bodily symptoms and physical limitations, but also with impairments in cognitive [45,46], social [47,48], and emotional [12,49,50] domains. In addition, many patients experience an altered sense of self [51,52] reduced overall functioning [14], and limited participation [53,54]. However, existing measures of HRQoL often focus on few isolated domains and pay less attention to domains such as cognition and perception of the self [13]. In contrast, the QOLIBRI is a disease-specific instrument that captures multiple aspects of HRQoL after TBI, encompassing physical, psychological (emotional and cognitive), social, self-image, and functional domains [14].

In contrast to the international study, this German-language sample was recruited in emergency and surgical wards of hospitals offering acute care for head injuries. Therefore, there were greater proportions of mild and elderly cases in this sample than in the international study, in which participants were predominantly recruited through rehabilitation centres. The variation in patients and the differences in the degree of severity presents an advantage for psychometric testing, although the relatively modest number of participants may have a limiting effect on the outcome of some analyses, e.g. the item response theory analyses, factor analyses, and structural equation modelling.

### Properties of the QOLIBRI

For international multi-centre studies, the equivalence of both the content and metrics of the instrument needs to be demonstrated [55–57]. The German translation of the QOLIBRI was based on a standardized procedure, including translation, back-translation, review, and cognitive debriefing [58,59]. Cross-cultural development included the demonstration of comparable metric properties for the whole sample.

#### Reliability

Results indicate that the psychometric properties of the German QOLIBRI are favourable. Internal consistencies and test-retest reliabilities for the individual German QOLIBRI subscales and the total score are good to very good, and they are slightly higher than in the international sample. The QOLIBRI scales are thus appropriate for analyses on a group level and most of them are also sufficiently reliable for assessing individuals. One concern with self-report instruments in TBI is the potential lack of insight that may be experienced by those with cognitive impairment. Experiences with diseases such as dementia suggest that subjective HRQoL judgments can be obtained reliably even in people with substantial cognitive impairments [60,61]. In the German sample, the internal consistency (i.e., Cronbach’s α) and test-retest reliability for participants with poorer cognitive performance were good to very good (0.79–0.91). The lowest reliability was recorded for the ‘‘Physical Problems” scale, an area in which some fluctuation might be expected. These findings indicate that even participants with lower cognitive performance were responding to the questionnaire rationally and consistently. However, we should add that this encouraging result should be considered preliminary as our study did not include a detailed neuropsychological assessment.

When the correlation between neuropsychological functioning and QoL have been investigated in the case of head injuries or other diseases of the central nervous system, the associations have usually been rather weak or absent [62]. It is notable, however, that in this German validation, correlations between the TICS and the QOLIBRI were quite high. One limitation is that the TICS is a rather crude instrument designed for screening cognitive functioning. HRQoL was also related to clinician ratings of cognitive problems, particularly problems of memory and attention. These findings indicate that impaired cognitive status is associated with poor HRQoL, i.e. low QOLIBRI scores. This result is promising and novel in TBI research and suggests a measurable relationship between these two classes of variables, and further research is warranted on this topic.

#### Structural validity

The conceptual model of HRQoL, on which the international QOLIBRI descriptive system was based, suggests a six-dimensional model with four ‘‘satisfaction” scales and two ‘‘bothered” scales. The German PCA and SEM analyses also support this six-factor structure of the QOLIBRI in a second-order latent variable model. As expected, the factor loadings were a little lower than in the international sample due to the small number of participants. The Rasch analysis also supports the structure of the QOLIBRI. The analysis identified only a small number of items that did not meet relatively conservative criteria for fit to a Rasch scale, and the extent of the deviation in these instances was minor. A cut-off of 1±0.4 is sometimes suggested for rating scales [63], and all items met this criterion. The QOLIBRI subscales generally had satisfactory Rasch reliability statistics, but the lower values for the “Emotions” and “Physical Problems” scales suggest that some caution is necessary when using these as individual measures.

#### Convergent validity

Concerning the validity of the German version of the QOLIBRI, the results show the expected pattern of relationships with other scales, thus confirming the validity of the scale. Particular patterns in the findings are consistent with expectations and comparable to, or slightly better than, the international studies (see Table 6): The SF-36 PCS has its highest correlation with the QOLIBRI “Physical Problems Scale” (.74), and a similar pattern is seen for “Comorbid Health Conditions” (.67). The SF-36 MCS correlates most highly with the “Emotions” (.69), and “Self” (.65) scales. The SWLS correlates most highly with “Self”(0.72) and “Daily Life and Autonomy” (0.66) scales. The HADS “Anxiety” scale correlates most strongly with the QOLIBRI “Emotions” scale (0.57), and HADS “Depression” with the “Self” scale (0.63). “Help needed with activities” correlates most with the “Daily Life” (0.63) and “Physical Problems” (0.51) scales. As would be expected, the strongest correlations with the GOSE are for “Physical Problems” (.56) and “Daily Life” (.51).

The SWLS and the QOLIBRI total score were strongly correlated in the sample, indicating substantial overlap in the constructs assessed. Both the SWLS and the QOLIBRI are satisfaction scales, and would thus be expected to overlap. The SWLS has been recommended as a core QoL measure for assessing TBI [64], and the question arises whether the QOLIBRI total adds anything to this well-established measure. Analysis using partial correlations with the GOSE demonstrates that the QOLIBRI contains information that is not captured by the SWLS. The relationships found with the GOSE are consistent with the idea that the SWLS reflects satisfaction with life in general, and the QOLIBRI more specifically tracks satisfaction with areas of life that are affected by a brain injury.

Another notable finding is the impact of disability, even moderate disability, on generic and TBI-specific HRQoL, as reported by the participants. The results of the comparison with the GOSE show that in each domain of the QOLIBRI, severely and moderately disabled TBI participants indicate lower HRQoL than those who had achieved a good level of recovery. It is particularly noteworthy that patients with moderate disabilities reported significantly lower HRQoL than those with a good recovery. It is still not always acknowledged, however, that even those with apparently mild injuries can suffer from moderate disability [65].

In the German as well as the international sample, the overall relationship between the GOSE and the QOLIBRI was only moderate, indicating that people can have a poor outcome on the GOSE and yet have good HRQoL, and vice versa. A new finding is that self-rated competency on the PCRS is quite strongly related to the QOLIBRI [66]. This supports the idea that the QOLIBRI is sensitive to common types of disability caused by head injury. There has been some debate over the relationship between functional outcome and HRQoL, with some authors claiming that the two are only weakly related. Brown and Gordon [67] have studied the relationship between disablement and quality of life, and found that less than 20% of the variance could typically be predicted by measures of disability. In contrast, the correlation between the total scores of the PCRS and the QOLIBRI here suggests that shared variance is over 50%. Brown and Gordon [67] argue that the measures of disablement with greater face validity are better predictors of QoL. In the present study, the person’s own view of their competence in functioning seems to be particularly strongly related to HRQoL.

The GOS/GOSE is currently the most popular outcome measure in acute brain injury trials; however, it is widely acknowledged that this assessment has limitations. It does not address some important domains, including cognition or self-perception [68,69]. As an assessment of functional outcome, it does not capture the subjective experience and self-report by the individual. In the field of brain injury, the interest in HRQoL has been partly encouraged by the failure of clinical trials in the acute stage to demonstrate clinical benefits using functional outcomes as a primary endpoint [70]. The development of this disease-specific HRQoL scale after TBI may therefore provide a good end point for clinical trials. It opens the possibility of constructing a composite multidimensional outcome assessment that covers both functional and HRQoL outcomes. Such a composite assessment would complement the picture of outcome after brain injury, and potentially provide a more sensitive tool for clinical trials.

Examining predictors of HRQoL, a regression analysis with the German data showed similar findings to those of the international sample, whereby the strongest predictors of QOLIBRI scores are aspects of the patient’s current status, and specifically emotional state, comorbid health conditions, and functional outcome, whereas demographic and clinical background factors have little if any influence. Given the average length of time since injury, it is not surprising that GCS played little if any role, and the current findings are in accordance with the work of others [14,45].

Previous reports of an association between QoL and emotional state [12,71–74], and loss of independence [75–77] are highlighted in our study in more detail. This has been underlined by the correlations between HADS anxiety and depression, POMS and self-reported health status symptoms, and the QOLIBRI. The association between depression and HRQoL is a ubiquitous finding, also reported in most other diseases, but it is generally acknowledged that HRQoL goes beyond simply assessment of emotional state [78,79].

### Limitations

The current study also has some limitations. Its intention was to validate the QOLIBRI in the German language, and it was therefore desirable to collect data from a large sample of brain injuries with a wide variety of clinical characteristics. The current cross-sectional design of the study was not designed to allow a straightforward analysis of recovery over time. In addition, given the wide range of intervals after the injury in our sample, our results do not allow inference of QoL after a certain mean interval, but are more indicative of “general” QoL after TBI. Another issue yet to be resolved by future research is the responsiveness of the QOLIBRI to change and susceptibility to response shift. The results show that the German QOLIBRI is sensitive to differences in outcome on the GOSE and other functional and patient characteristics. Moreover, due to the scope of our study it was impossible to investigate the impact of impairment of self-awareness or cognition on generic and TBI-specific HRQoL self-rating in more detail. However, relying on the instruments we used we were able to establish, for the German as well as for the total sample, that level of cognitive function did not affect the reliability of reporting [22,23]. For detailed information on the impact of impaired self-awareness on HRQoL see [66,80].

### Conclusion & outlook

In conclusion, the psychometric properties of the German QOLIBRI suggest that it is a practical and reliable instrument that can be considered for use in studies examining HRQoL after TBI in clinical as well as in research settings. The QOLIBRI offers a profile of HRQoL in domains that are relevant for TBI, as well as a summary score. It provides an assessment of TBI-specific HRQoL that potentially complements functional outcome measures such as the GOS or GOSE and measures of social participation, general wellbeing and subjective health status.

Development of this instrument was encouraged by many people working in post-acute brain injury management, and especially in rehabilitation. Currently the English-language version of the QOLIBRI has been downloaded more than 1000 times by research and rehabilitation centres; project outlines describe the use of the QOLIBRI, for example, to measure the effectiveness of cognitive and functional therapy. Outcome measures assessing TBI-specific HRQoL may contribute to quality control in short- and long-term care, medical decision-making, and rehabilitation planning. In clinical use in the rehabilitation setting, it can help to identify and set appropriate goals for therapy. The QOLIBRI covers several domains currently missing from TBI-specific item bank initiatives such as PROMIS or NEURO-QOL, and these systems may profit from including items from the QOLIBRI [81].

The final descriptive system of the German QOLIBRI provides both a comprehensive HRQoL profile across six domains of life, and a total index of HRQoL after TBI. When used in evaluation studies, shifts in individual domains will reflect areas of life where gains or losses following intervention or treatment are noticeable. In contrast, where an index is required, the QOLIBRI total score can be used to assess the impact of treatments on HRQoL. In addition, where a very short instrument is needed, we have developed an overall screening measure, the German QOLIBRI-OS, with six items and good psychometric properties covering the most important areas of HRQoL [82].

## Supporting information

S1 File Raw data. (POR)
